# Are Imaging Evaluations of Soft-Tissue Masses Before Referral to a Specialized Center Being Performed Properly? A Systematic Review

**DOI:** 10.3390/cancers16233935

**Published:** 2024-11-24

**Authors:** Min Wook Joo, Chan Jin Park, Yong-Suk Lee, Yoon Joo Cho, Nicholas Matthew Bernthal, Seul Ki Lee, Hyunho Kim, Joo Hwan Lee, Sung Hwan Kim, Yang-Guk Chung

**Affiliations:** 1 Department of Orthopaedic Surgery, St. Vincent’s Hospital, College of Medicine, The Catholic University of Korea, 222 Banpo-daero, Seocho-gu, Seoul 06591, Republic of Korea; cjinpark@gmail.com (C.J.P.); choyj8908@gmail.com (Y.J.C.); 2 Department of Orthopaedic Surgery, University of California Los Angeles, Los Angeles, CA 90095, USA; nbernthal@mednet.ucla.edu; 3 Department of Orthopedic Surgery, Incheon St. Mary’s Hospital, College of Medicine, The Catholic University of Korea, 222 Banpo-daero, Seocho-gu, Seoul 06591, Republic of Korea; maerie@naver.com; 4 Department of Radiology, St. Vincent’s Hospital, College of Medicine, The Catholic University of Korea, 222 Banpo-daero, Seocho-gu, Seoul 06591, Republic of Korea; beneffy@catholic.ac.kr; 5 Division of Medical Oncology, Department of Internal Medicine, St. Vincent’s Hospital, College of Medicine, The Catholic University of Korea, 222 Banpo-daero, Seocho-gu, Seoul 06591, Republic of Korea; h2kim@catholic.ac.kr; 6 Department of Radiation Oncology, St. Vincent’s Hospital, College of Medicine, The Catholic University of Korea, 222 Banpo-daero, Seocho-gu, Seoul 06591, Republic of Korea; rainonly@catholic.ac.kr (J.H.L.); kimandre@catholic.ac.kr (S.H.K.); 7 Department of Orthopedic Surgery, Seoul St. Mary’s Hospital, College of Medicine, The Catholic University of Korea, 222 Banpo-daero, Seocho-gu, Seoul 06591, Republic of Korea; ygchung@catholic.ac.kr

**Keywords:** soft-tissue neoplasm, referral and consultation, diagnostic imaging, ultrasonography, magnetic resonance imaging

## Abstract

Background/Objectives: Since the initial approach towards the clinical presentation of soft-tissue masses is challenging for frontline physicians, some countries use clinical practice guidelines. Proper imaging work-up is crucial to differentiate between soft-tissue tumors. Recently, ultrasonography and magnetic resonance imaging (MRI) have been widely used. But there is some controversy about whether pre-referral evaluations are being conducted properly. Thus, this study aims to assess whether the ultrasonography investigation of soft-tissue masses prior to referral to the musculoskeletal tumor center is being performed adequately in terms of indications, diagnostic accuracy, and referral interval, and to evaluate whether the pre-referral MRI for soft-tissue masses is being conducted reasonably concerning indications, imaging protocol, reporting, diagnostic accuracy, and cost-effectiveness. Methods: The study protocol was registered. We followed the Preferred Reporting Items for Systematic Reviews and Meta-Analyses checklist. We performed a database search of the MEDLINE, Embase, and Cochrane Library. Then, two authors reviewed the studies, and the third author resolved any disagreement between them. A total of nine studies were included. The Risk Of Bias In Non-Randomized Studies-of Interventions was applied. Relevant data were extracted by two authors. Results: This review showed that the way that the imaging investigations are performed in non-specialized centers before referral was generally considered inappropriate. Conclusions: Frontline physicians should regard the alarm symptom as an indication for advanced imaging evaluation. Education and certification may be required for ultrasonography. MRI should be performed and interpreted in a specialized center or by a specialist with relevant expertise. Guidance may help reduce inappropriate imaging.

## Simple Summary

Although physicians often encounter patients with soft-tissue masses, the initial approach towards the clinical presentation is never straightforward for non-specialists, especially in terms of differentiating between malignant and other aggressive musculoskeletal tumors. As most doctors encounter very few sarcoma patients in their practice, some clinical guidelines on soft-tissue masses have been implemented. Recently, ultrasonography and MRI have been widely used for soft-tissue masses, even in referring hospitals, but there is some controversy over the appropriateness of such pre-referral evaluations. This study showed that the way that imaging investigations are performed in non-specialized centers prior to referral was generally regarded as improper. Frontline physicians should comprehend alarm symptoms as an indication for advanced imaging evaluation. Education and certification may be required for ultrasonography. MRI should be performed and interpreted by specialists with relevant expertise or in a specialized center. Guidance may help reduce inappropriate imaging.

## 1. Introduction

Frontline physicians often encounter patients with soft-tissue masses. Nevertheless, the initial diagnostic approach is challenging for many clinicians as it can be one of a myriad of diseases, such as infectious, traumatic, or tumorous conditions [1]. Especially, a considerable overlap in the clinical presentation of benign and malignant musculoskeletal tumors, and a lack of relevant knowledge, can result in undesirable decisions by non-specialists in terms of evaluation, referral, and treatment [2]. A malignant soft-tissue tumor is rare. According to the Annual Report of Korea National Cancer Registry in 2020 [3], the number of malignant neoplasms of peripheral nerves and the autonomic nervous system, and other connective and soft tissue, classified under codes C47 and C49 in the International Classification of Disease (ICD)-10, was 1254 (720 in men and 534 in women), accounting for 0.5% of the total 61 malignancies [3]. Most doctors encounter very few patients with the disease in their careers. Thus, some countries use clinical practice guidelines for soft-tissue masses, such as the SSG (Scandinavian Sarcoma Group), SEOM (Spanish Society of Medical Oncology), and NICE (National Institute for Health and Care Excellence) guidelines [4,5,6]. The guidelines universally recommend further evaluation for potential malignancy in a mass larger than 4 to 5 cm, or deeply seated [4,5,6].

When a suspicious mass is encountered, proper imaging work-up is crucial to differentiate between malignant or other aggressive soft-tissue tumors [7,8]. Ultrasonography is often used to assess soft-tissue masses as it is flexible, convenient, and easy to access [9]. It can not only show the dynamic characteristics of lesions but also distinguish between cystic and solid components [9]. Magnetic resonance imaging (MRI) is an advanced diagnostic modality for musculoskeletal tumors suspected of being malignant [10]. It is a technique of choice for local staging and valuable in determining lesion characteristics [11]. Recently, advanced imaging modalities have been widely used for soft-tissue masses, even in the referring institutions [7]. According to a report of the Korea Health Industry Development Institute, clinics had 21,697 ultrasonography units and 283 MRI units in 2020, hospitals had 4662 ultrasonography units and 759 MRI units, general hospitals had 5317 ultrasonography units and 505 MRI units, and tertiary hospitals had 3080 ultrasonography units and 195 MRI units, respectively [12]. But there is some controversy about whether imaging evaluations in the non-specialized centers before referral are being conducted properly [7,9,10,11,13,14,15,16,17,18].

Thus, we performed a systematic review to address the following questions: (1) Is the ultrasonography investigation of soft-tissue masses before referral to the musculoskeletal tumor center being performed adequately in terms of indication, diagnostic accuracy, and referral interval? (2) Is pre-referral MRI for soft-tissue masses being conducted reasonably in terms of indication, imaging protocol, reporting, diagnostic accuracy, and cost-effectiveness?

## 2. Materials and Methods

The study protocol was prospectively registered in the International Prospective Register of Systematic Reviews, with registration number CRD42023455652. We followed the Preferred Reporting Items for Systematic Reviews and Meta-Analyses checklist.

### 2.1. Search Strategy

We comprehensively performed a database search of the MEDLINE, Embase, and Cochrane Library from the inception date to 24 September 2023. Electronic search was performed based on indexing terms in each database, such as Medical Subject Heading (MeSH) and Emtree, and free-text terms. The main keywords in our searches were “soft-tissue neoplasm”, “referral and consultation”, and “diagnostic imaging” (Supplement S1). Articles written in languages other than English were excluded.

### 2.2. Eligibility Criteria and Selection Process

Studies discussing pre-referral ultrasonography and MRI for evaluation of soft-tissue masses were included, while studies that discussed the other modalities were excluded. Additional exclusion criteria included articles discussing specific histologic diagnoses, tumors arising in specific body parts, carcinomas, or bone tumors only. Acceptable studies were retrospective, and prospective descriptive, ones. Case reports, review articles, letter to the editor, abstracts without available full text, and expert opinions were excluded. The authors (M.W.J. and C.J.P.) manually and independently reviewed studies for eligibility. These reviewers scrutinized the titles and abstracts to assess the relevance of articles. The same reviewers read the full text of all pertinent articles, and eligible articles were included. The third author (Y.J.C.) resolved any disagreement between the two reviewers in the selection process.

**Figure 1:**
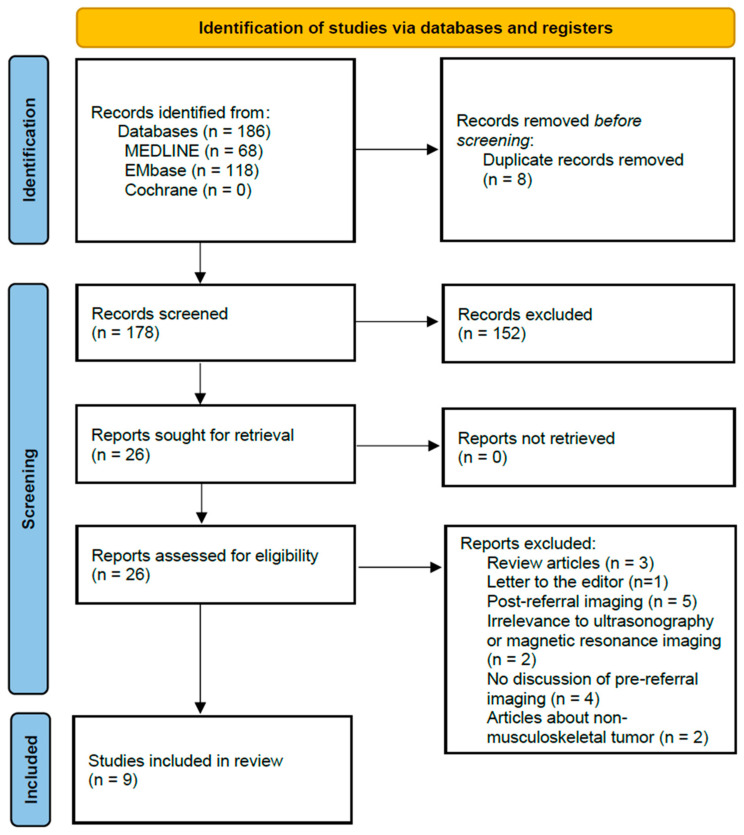
The Preferred Reporting Items for Systematic Reviews and Meta-Analyses (PRISMA) flow diagram on the process of study selection.

The electronic search yielded 186 studies from these three databases. After duplicates were discarded, 178 papers remained. Then, after the title and abstract review, 26 pertinent articles were left. A final full-text review confirmed that these studies met the inclusion criteria. None of the studies were added through citation tracking, and articles on preprint servers were not considered. The full-text review excluded three studies because they were review articles and one study because it was a letter to the editor. Five articles were removed since they only included post-referral imaging. Two were excluded because they did not include ultrasonography or MRI in the diagnostic work-up. Four were removed for not discussing pre-referral imaging. Lastly, two were excluded because they discussed unrelated anatomical sites. A total of nine studies were included for final review (Figure 1).

### 2.3. Data Synthesis

Relevant data were extracted independently by two authors (M.W.J. and C.J.P.). The outcome measures were propriety of indications, diagnostic accuracy and referral interval for ultrasonography, and appropriateness of indications, technical fidelity of the imaging protocol, faithfulness of reporting, diagnostic precision and cost-effectiveness for MRI.

## 3. Results

### 3.1. Study Characteristics

These nine studies used either ultrasonography or MRI as a pre-referral examination (Table 1 and Table 2). Six articles discussed MRI, one ultrasonography, and two both. All studies included soft-tissue tumors. Seven articles were retrospective in design, while two were prospective.

### 3.2. Risk of Bias

**Figure 2:**
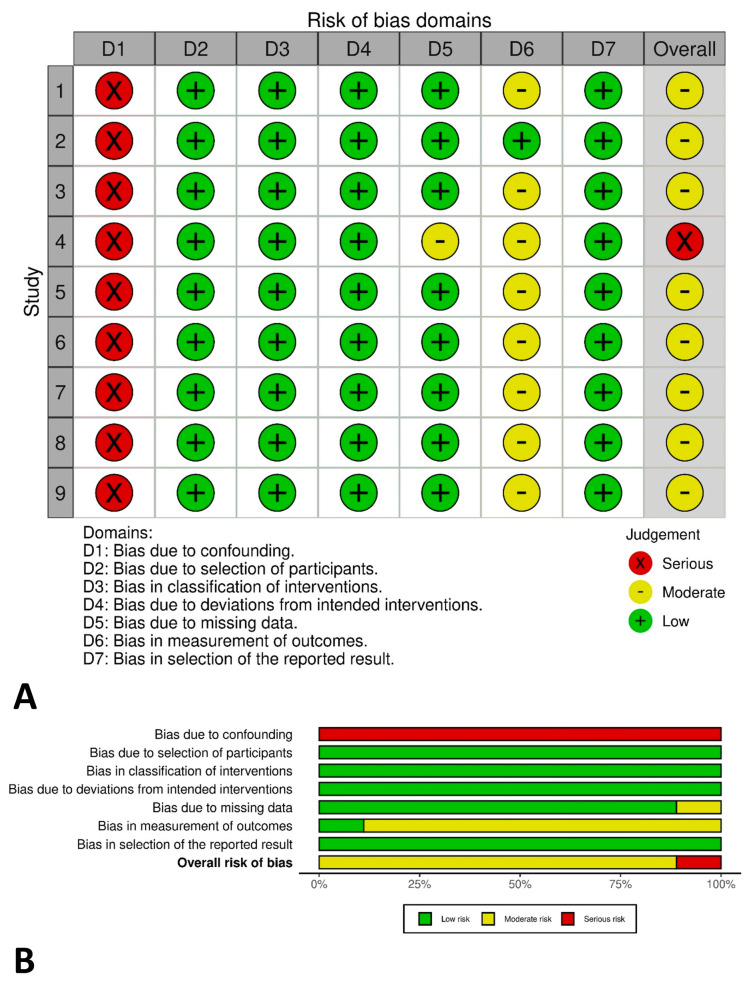
The risk of bias graph by the Risk of Bias in Non-Randomized Studies of Interventions (ROBINS-I) tool. (**A**) Traffic light plot. (**B**) Summary plot.

Since all included studies were non-randomized, the Risk Of Bias In Non-Randomized Studies of Interventions tool was applied. Two authors (M.W.J. and C.J.P.) independently performed the analysis, and any disagreement was sorted by the third author (Y.J.C.). The risk of bias was graded as moderate in eight studies and serious in one study, respectively (Figure 2).

### 3.3. Ultrasonography

In 371 patients referred to eight centers with eight fellowship-trained orthopedic oncologists across the United States (US), 21 pre-referral ultrasonograms were performed and 16 (76%) pre-referral ultrasonograms were not thought to be those that the treating specialists would generally perform in a given situation or would aid in deciding the diagnosis or setting up a management plan [18].

Among 397 patients with any soft-tissue mass and one or more specific characteristics suspicious of malignancy referred under the two-week wait rule to the Cambridge Sarcoma Diagnostic Clinic of the Cambridge University Hospitals NHS Foundation Trust between January 2013 and December 2014, malignancy was not diagnosed in 64 patients who underwent ultrasonography alone before referral. The characteristics for referrals were size larger than 5 cm, pain, increase in size, sub-fascial location, and recurrence after prior excision [13]. Among 175 patients referred from centers throughout the North Island of New Zealand to a multidisciplinary team at Middlemore Hospital after the ultrasonography examination between 1999 and 2009, 60 patients were categorized into the non-benign group in the original report [9]. Of them, thirty-five patients were correctly identified as having non-benign lesions, seven as having benign lesions, and eighteen as having lesions not specific in pathologic diagnosis. For 144 patients, recommendations for further evaluation, either implied by a non-benign diagnosis or explicitly in the form of a suggestion for MRI, follow-up study, or seeking a specialist surgeon’s opinion, were offered in the ultrasonography report. Of them, 92 patients were confirmed to have a benign lesion. On the other hand, final pathology was non-benign in eight of thirty-one patients with no recommendation; the effective false negative rate was 13% (95% confidence interval 6% to 24%); and the sensitivity, specificity, positive predictive value, negative predictive value, positive likelihood ratio, and negative likelihood ratio were 87%, 20%, 36%, 72%, 1.1, and 0.67, respectively [9].

In the above eight patients with a non-benign pathologic diagnosis confirmed after incorrect recommendation, the median delay in reaching the definitive diagnosis was 1.5 months (range, 0 to 10 months) [9].

### 3.4. Magnetic Resonance Imaging

Among 371 patients transferred to eight orthopedic oncologists across the US, 263 patients underwent MRI before referral and 46 (17%) MRI studies were not deemed to be those that cancer specialists would usually consider for a given presentation or to help determine the imaging diagnosis or plan treatment [18]. Among the patients with a suspected bone or soft-tissue tumor referred to an orthopedic oncology practice, MRI scans were observed in 76 patients [17]. Twenty-three of forty-one (56%) MRI scans in the group with benign bone tumors or non-neoplastic conditions, and three of thirty-five scans in the group with malignant bone tumors or soft-tissue tumors were not the study part of proper work-up for the diagnosis or were unhelpful in establishing the diagnosis and management. Meanwhile, 320 pre-referral MRI examinations were obtained in 920 new patients suspected of having a bone or soft-tissue tumor transferred to the musculoskeletal oncology clinic between January 2009 and December 2010, and eight examinations were determined as not to be indicated [10]. The indications were a primary bone sarcoma, biopsy-proven soft-tissue sarcomas, soft-tissue masses larger than 5 cm in diameter, sub-fascial soft-tissue masses, pain, and growth.

Among 125 consecutive patients referred to a specialist musculoskeletal oncology unit for evaluation of soft-tissue masses between September 2018 and May 2020, 93 MRI studies were obtained prior to referral, including varied combinations of imaging protocols in contrast to the studies performed following referral [14]. Among 50 consecutive MRI examinations performed in patients referred to orthopedic oncologists at The London Bone and Soft-Tissue Tumour Unit over a one-year period, intravenous contrast medium was administered in 19 cases [11]. Among 320 pre-referral MRI scans in 920 patients transferred to the musculoskeletal oncology clinic from 2009 to 2010, eight MRI scans lacked contrast enhancement and four patients had partial tumor imaging [10]. In 371 patients referred to eight sarcoma centers, lack of contrast and inadequate visualization of the tumor were observed in 11 and six of the 236 pre-referral MRI examinations [18].

Among MRI scans in 14 patients with a soft-tissue tumor referred to orthopedic oncologists over a one-year period, information on the relationship to the neurovascular bundle and underlying bone, anatomical location, and dimensions was included in MRI reports of two (14%), four (29%), fourteen (100%), and nine (64%) patients, respectively [11].

Among three-hundred and ninety-seven patients with any soft-tissue mass and one or more characteristics suspicious of malignancy referred to the Cambridge Sarcoma Diagnostic Clinic in 2014, malignancy was diagnosed in one out of sixty-one patients, and in one out of one-hundred and thirteen who had pre-referral MRI alone, and MRI with ultrasonography, respectively [13]. From sixty-nine patients with a pre-referral diagnosis of possible soft-tissue sarcoma on MRI among one-hundred and twenty-five referred from 2018 to 2020, only six patients were diagnosed with sarcoma while four were diagnosed with an intermediate-grade tumor [14].

A total of 210 MRI scans were performed in 192 of 298 consecutive new patients referred to a tertiary center for evaluation of a suspected bone or soft-tissue neoplasm during a course of three months [7]. Among them, 56 scans were not indicated or were of poor technical quality. The indications were size greater than 5 cm, distality or involvement of the wrist or ankle, history of growth, recent pain and no response to non-surgical treatment, neurologic symptoms, the necessity of intra-articular evaluation based on abnormal clinical examination, and bone destruction. Technical inappropriateness was defined as failure to image the entire lesion, improper sequences and reconstructions, and a lack of intravenous contrast.

In about 40 patients with a soft-tissue tumor referred to the supraregional center in the United Kingdom between October 1997 and December 1998, almost all MRIs had to be repeated because they provided no appropriate information for staging or planning management [15]. Among 320 pre-referral MRI scans in 920 patients with suspected musculoskeletal tumors, 12 scans were repeated due to technical inadequacy [10]. In 371 patients referred to sarcoma centers, MRI evaluations were repeated owing to lack of contrast and inadequate visualization of the tumor in 17 of 236 pre-referral MRI examinations [18].

## 4. Discussion

Although physicians often encounter patients with soft-tissue masses, the initial approach towards the clinical presentation is never simple for non-specialists, especially in terms of differentiating the malignant or other aggressive musculoskeletal tumor [19]. As most doctors encounter very few sarcoma patients in their practice, some clinical guidelines on soft-tissue masses have been implemented [4,5,6]. Recently, ultrasonography and MRI have been widely used for soft-tissue masses, even in the referring hospitals [7], but there is some controversy over the appropriateness of such pre-referral evaluation [7,9,10,11,13,14,15,16,17,18]. In this study, pre-referral ultrasonography and MRI evaluations conducted in non-specialized institutions were generally regarded as improper in terms of indication, protocol, reporting, accuracy, referral delay, and cost-effectiveness [7,9,10,11,13,14,15,16,17,18].

There are some limitations to our review. Studies on bone tumors were carefully excluded in the selection process, but the literature that covered the results for soft-tissue and bone tumors under the comprehensive scope of a musculoskeletal tumor without separation was included after deliberation [7,10,17,18]. Although patients with soft-tissue masses are referred to a specialized center, the final diagnosis might include both soft-tissue and bone tumors. It should be considered that ultrasonography is commonly used in soft-tissue tumors rather than in bone tumors [9,20], and a plain radiograph is recommended as the first evaluation of a bone tumor prior to MRI [7,13,21]. Second, the lack of overlapping metrics in the literature resulted in the narrative form of the results without quantitative data analysis, making it difficult to take in the lines at a glance [22,23]. Third, since the evidence is based on studies of referred cases, this review may not reveal the real practice of conducting imaging investigations in referring institutions and may underestimate the rate of inappropriate evaluations. Lastly, because of the large differences in medical fees between countries and institutions [24], the cost-effectiveness could not be clearly reflected simply by the actual cost. Therefore, the repetition rate of advanced imaging tests in the referred center was reviewed [10,18].

A battery of advanced imaging studies may be ordered without regard to a logical flow based on the differential diagnosis [17]. The European Society of Skeletal Radiology (ESSR)-approved guidelines for the diagnostic imaging of soft-tissue tumors [8] have suggested that some information on the patient’s history and clinical features should be available to the radiologist. The history includes recent trauma, anticoagulant administration, symptom duration, growth and size change in the lesion, underlying oncologic disease, and prior surgery. The features were pain, consistency, mobility, skin or vascular alteration, and multiplicity. Meanwhile, adaptations of the features of concern were recommended. As pain was not a reliable factor with 27% sensitivity and 66% specificity for possible malignancy, its removal from an urgent referral form was proposed [25]. Likewise, a simple guideline for efficient referral of the soft-tissue mass [26] does not include pain or tumor growth because most soft-tissue masses are painless and incidentally noted. Frontline physicians should not forget such recommendations on the classical alarm symptom as an indication for advanced imaging evaluations [4,5,6].

The following are suggested as technical standards for ultrasonography [8]: the equipment should meet quality assurance criteria, images should be stored in a picture archiving and communication system, and a written report should be provided for every examination. Advanced scanners should be employed, equipped with high-resolution transducers, with frequency adjusted based on lesion depth, and color/power Doppler capabilities. An extended field of view and compound imaging are preferred. If no mass is initially found, harmonic imaging with frequency variation may be helpful for detecting echo-poor solid masses. While ultrasonography can be a safe and effective diagnostic triage tool for the assessment of soft-tissue masses [27], it holds an inherent risk of misdiagnosis due to the relative lack of knowledge of soft-tissue sarcomas and other aggressive tumors in the examiner at the referring institutions [9,13]. Ideally, ultrasonography should be performed by a physician or a radiologist with proven experience in sonographic soft-tissue lesion assessment [8]. Education and certification may be required for referrers to understand its limitations and pitfalls, which should be highlighted especially if they are not trained in the management of soft-tissue masses [9].

It is also recommended to preferably perform MRI in a specialized center or by a radiologist with sufficient relevant expertise [8]. The ESSR guideline [8] has suggested technical MRI requirements, such as 1.5 or 3-T preferential field strength of the scanner, a cutaneous marker, and the field of view as large as necessary to cover the entire lesion, perilesional edema, a layer of adjacent normal tissue, and non-palpable lesions. Slice thickness should not exceed 4 mm. A lesion should be estimated in three dimensions, and an external bony landmark should be imaged in at least one sequence. The use of intravenous contrast is recommended, and post-contrast sequences should be performed in two planes. Diffusion-weighted sequences may be included to help characterize the lesion [14]. On the MRI report, the anatomical location and extension of a lesion in relation to the surrounding tissues and external landmark, relation to the fascia, and details of lesion morphology should be described. Nevertheless, a study [28] concluded that most radiologists practicing in a region did not follow the ESSR guidelines, based on the comparison of 48 patients before and 55 patients before and after the introduction of the guidelines. When the guidelines were applied, it was only followed for the recommended MRI sequences, while all other technical requirements were largely overlooked. Another study [29] on 126 patients from a sarcoma center also identified significant deviations of MRI protocols and reports from the ESSR guidelines. Recently, the ESSR suggested a diagnostic algorithm for local imaging of soft-tissue masses across different clinical scenarios [27].

The overutilization of healthcare resources is a controversial issue [10,30]. Patients with soft-tissue masses might undergo costly improper advanced imaging studies before referral to a specialized center [17]. Transferring the patients before performing the examinations can reduce these costs [7,17]. Early referral will achieve significant cost savings, especially for patients with benign or non-neoplastic lesions [17]. The education of frontline providers regarding the judicious use of advanced imaging in soft-tissue masses may help reduce inappropriate imaging [18]. Nevertheless, this systematic review does not advocate for the total prohibition of pre-referral evaluations, but rather it suggests the minimization of inappropriate tests and the initial pursuance of accurate diagnostic performances [18]. Instead, it is important to guide the use of imaging modalities when clinically indicated [18].

## 5. Conclusions

This review showed that ultrasonography and MRI investigations performed in non-specialized centers before referral were generally considered inappropriate in terms of indication, protocol, reporting, accuracy, referral delay, and cost-effectiveness. Frontline physicians should comprehend the alarm symptom as an indication for advanced imaging evaluation. Education and certification may be required for ultrasonography. MRI should be performed and interpreted in a specialized center or by a specialist with relevant expertise. Guidance may help reduce inappropriate imaging.

## Figures and Tables

**Table 1:** Characteristics of studies on ultrasonography included in the final review.

Study (Year)	Country	Type of Study	Population	Period	Imaging Investigation	Main Results
Kwok et al. [9] (2012)	New Zealand	Retrospective	Patients referred to multidisciplinary team	1999–2009	USG (n = 175)	Correct diagnosis of non-benign pathology = 35/60 No recommendations for further management in non-benign pathology 8/31: median 1.5-month delay in definitive diagnosis
Miller et al. [18] (2015)	US	Prospective	Patients referred to fellowship-trained orthopedic oncologist	-	USG (n = 21)	Unhelpful USG = 16/21 (76%)
Szucs et al. [13] (2016)	UK	Retrospective	Patients referred to sarcoma diagnostic clinic	2013–2014	USG (n = 64)	Malignancy USG = 0%

US = United States; UK = United Kingdom; and USG = ultrasonography.

**Table 2:** Characteristics of studies on magnetic resonance imaging included in the final review.

Study (Year)	Country	Type of Study	Population	Period	Imaging Investigation	Main Results
Saifuddin et al. [11] (2000)	UK	Retrospective	Patients referred to orthopedic oncologists	-	MRI (n = 50)	Non-enhanced MRI = 62% Information in reports for soft-tissue tumors (n = 14) - Relationship to neurovascular bundle = 14% - Anatomical location = 100% - Dimensions = 64% - Relationship to underlying bone = 29%
Aboulafia et al. [17] (2002)	US	Prospective	Patients referred to orthopedic oncology practice	-	MRI (n = 76)	Unnecessary MRI = 26/76 (34.2%) - 23/41 (56%) in group with benign bone tumors or non-neoplastic conditions - 3/35 (9%) in group with malignant bone tumors or soft-tissue tumors
Ashwood et al. [15] (2003)	UK	Retrospective	Patients referred to supraregional bone and soft-tissue tumor service	October 1997–December 1998	MRI (n ≈ 40)	Repetition of almost all MRI
Martin et al. [10] (2012)	US	Retrospective	Patients referred to musculoskeletal oncology clinic	January 2009–December 2010	MRI (n = 320)	Inappropriate MRI = 20 (6.2%) - Unnecessary MRI = 8 - Non-contrast MRI = 8 - MRI without full extent of tumor = 4 - Repetition of MRI = 12
Miller et al. [18] (2015)	US	Prospective	Patients referred to fellowship-trained orthopedic oncologist	-	MRI (n = 263)	Unhelpful MRI = 46/236 (17%) Repetition of MRI = 17/236 (6%) - Lack of contrast = 11 - Inadequate visualization of tumor = 6
Nystrom et al. [7] (2015)	US	Retrospective	Patients presenting to tertiary care referral center	-	MRI (n = 210)	Inappropriate MRI = 26.7%
Szucs et al. [13] (2016)	UK	Retrospective	Patients referred to sarcoma diagnostic clinic	2013–2014	MRI (n = 61) USG + MRI (n = 113)	Malignancy - MRI = 1.6% - USG + MRI = 0.9%
Reid et al. [14] (2020)	UK	Retrospective	Patients referred to a specialist musculoskeletal oncology unit	September 2018–May 2020	MRI (n = 93)	Varied combination of imaging protocols in pre-referral MRI Pre-referral imaging diagnosis of possible sarcoma: final diagnosis = 59.5%:5.2%

US = United States; UK = United Kingdom; USG = ultrasonography; and MRI = magnetic resonance imaging.

## Data Availability

The data presented in this study are available in Pubmed, Embase, and Cochrane.

## Supplementary Materials

The following supporting information can be downloaded at: https://www.mdpi.com/article/10.3390/cancers16233935/s1, Supplement S1: Search Strategies.

## References

[1] Kang H.G. (2015). Diagnoses and Approaches of Soft Tissue Tumors for Orthopaedic Non-Oncologists. J. Korean Orthop. Assoc..

[2] Crombé A., Kind M., Fadli D., Miceli M., Linck P.-A., Bianchi G., Sambri A., Spinnato P. (2023). Soft-tissue sarcoma in adults: Imaging appearances, pitfalls and diagnostic algorithms. Diagn. Interv. Imaging.

[3] Korea Central Cancer Registry, National Cancer Center (2022). Annual Report of Cancer Statistics in Korea in 2020.

[4] SSG Guidelines for Referral of Patients with Soft Tissue Tumors of the Extremities and Trunk Wall. https://www.ssg-org.net/treatment-protocols-and-recommendations/ongoing.

[5] López-Pousa A., Broto J.M., Trufero J.M., Sevilla I., Valverde C., Alvarez R., Alvarez J.A.C., Jurado J.C., Hindi N., Del Muro X.G. (2016). SEOM Clinical Guideline of management of soft-tissue sarcoma. Clin. Transl. Oncol..

[6] National Institute for Health and Clinical Excellence (2006). Guidance on Cancer Services: Improving Outcomes for People with Sarcoma.

[7] Nystrom L.M., Reimer N.B., Dean C.W., Bush C.H., Scarborough M.T., Gibbs C.P. (2015). Evaluation of imaging utilization prior to referral of musculoskeletal tumors: A prospective study. J. Bone Jt. Surg. Am..

[8] Noebauer-Huhmann I.M., Weber M.A., Lalam R.K., Trattnig S., Bohndorf K., Vanhoenacker F., Tagliafico A., van Rijswijk C., Vilanova J.C., Afonso P.D. (2015). Soft Tissue Tumors in Adults: ESSR-Approved Guidelines for Diagnostic Imaging. Semin. Musculoskelet. Radiol..

[9] Kwok H.C., Pinto C.H., Doyle A.J. (2012). The pitfalls of ultrasonography in the evaluation of soft tissue masses. J. Med. Imaging Radiat. Oncol..

[10] Martin C.T., Morcuende J., Buckwalter J.A., Miller B.J. (2012). Prereferral MRI use in patients with musculoskeletal tumors is not excessive. Clin. Orthop. Relat. Res..

[11] Saifuddin A., Twinn P., Emanuel R., Cannon S.R. (2000). An audit of MRI for bone and soft-tissue tumours performed at referral centres. Clin. Radiol..

[12] Dan B., Kim S.Y., Hwang S.E., Yoo K.H. (2021). Analysis of the Current Status of Medical Device Use by Domestic Medical Institutions: Focusing on HIRA’s Medical Equipment Possession and Treatment Material Claim Statistics.

[13] Szucs Z., Davidson D., Wong H.H., Horan G., Bearcroft P.W., Grant I., Grimer R., Hopper M.A., Hatcher H., Earl H.A. (2016). Comprehensive Single Institutional Review of 2 Years in a Designated Fast-Track Sarcoma Diagnostic Clinic Linked with a Sarcoma Specialist Advisory Group: Meeting the Target but Failing the Task?. Sarcoma.

[14] Reid C., Saifuddin A. (2021). A review of paediatric soft tissues masses referred to a tertiary musculoskeletal sarcoma centre. Br. J. Radiol..

[15] Ashwood N., Witt J.D., Hallam P.J., Cobb J.P. (2003). Analysis of the referral pattern to a supraregional bone and soft tissue tumour service. Ann. R. Coll. Surg. Engl..

[16] Dyrop H.B., Vedsted P., Rædkjær M., Safwat A., Keller J. (2017). Imaging investigations before referral to a sarcoma center delay the final diagnosis of musculoskeletal sarcoma. Acta Orthop..

[17] Aboulafia A.J., Levin A.M., Blum J. (2002). Prereferral evaluation of patients with suspected bone and soft tissue tumors. Clin. Orthop. Relat. Res..

[18] Miller B.J., Avedian R.S., Rajani R., Leddy L., White J.R., Cummings J., Balach T., MacDonald K. (2015). What is the use of imaging before referral to an orthopaedic oncologist? A prospective, multicenter investigation. Clin. Orthop. Relat. Res..

[19] Chun Y.S., Song S.H. (2019). Diagnostic Approach to a Soft Tissue Mass. J. Korean Orthop. Assoc..

[20] Yoo H.J. (2014). Sonographic Features of Common Soft Tissue Masses in the Extremities. J. Korean Orthop. Assoc..

[21] Shin D.-S., Ryu S.-M., Park C.-H. (2015). The Diagnostic Strategy for Malignant Bone Tumors. J. Korean Orthop. Assoc..

[22] Ng M.K., Magruder M.L., Heckmann N.D., Delanois R.E., Piuzzi N.S., Krebs V.E., Mont M.A. (2024). How-To Create an Orthopaedic Systematic Review: A Step-by-Step Guide Part I: Study Design. J. Arthroplast..

[23] Ng M.K., Magruder M.L., Piuzzi N.S., Heckmann N.D., Delanois R.E., Krebs V.E., Mont M.A. (2024). How-To Create an Orthopaedic Systematic Review: A Step-by-step Guide Part II: Study Execution. J. Arthroplast..

[24] Yoon J.H., Kwon I.H., Park H.W. (2024). The South Korean health-care system in crisis. Lancet.

[25] Smolle M.A., Leithner A., Grimer R.J. (2015). Evaluating the British sarcoma referral form. Ann. R. Coll. Surg. Engl..

[26] Styring E., Billing V., Hartman L., Nilbert M., Seinen J.M., Veurink N., Vult von Steyern F., Rydholm A. (2012). Simple guidelines for efficient referral of soft-tissue sarcomas: A population-based evaluation of adherence to guidelines and referral patterns. J. Bone Jt. Surg. Am..

[27] Noebauer-Huhmann I.M., Vanhoenacker F.M., Vilanova J.C., Tagliafico A.S., Weber M.A., Lalam R.K., Grieser T., Nikodinovska V.V., de Rooy J.W.J., Papakonstantinou O. (2024). Soft tissue tumor imaging in adults: European Society of Musculoskeletal Radiology-Guidelines 2023-overview, and primary local imaging: How and where?. Eur. Radiol..

[28] Korthaus A., Weiss S., Barg A., Salamon J., Schlickewei C., Frosch K.-H., Priemel M. (2022). Clinical Routine and Necessary Advances in Soft Tissue Tumor Imaging Based on the ESSR Guideline: Initial Findings. Tomography.

[29] Weiss S., Korthaus A., Baumann N., Yamamura J., Spiro A.S., Lübke A.M., Frosch K.-H., Schlickewei C., Priemel M. (2021). Musculoskeletal Soft-Tissue Sarcoma: Quality Assessment of Initial MRI Reports Shows Frequent Deviation from ESSR Guidelines. Diagnostics.

[30] Baicker K., Obermeyer Z. (2022). Overuse and Underuse of Health Care: New Insights from Economics and Machine Learning. JAMA Health Forum.

