# Supplementary material for: Are Imaging Evaluations of Soft-Tissue Masses Before Referral to a Specialized Center Being Performed Properly? A Systematic Review
Source: Cancers (Basel). 2024 Nov 24;16(23):3935. doi: 10.3390/cancers16233935 (PMC11640488; doi:10.3390/cancers16233935)
Supplement: Supplementary file 1 [file cancers-16-03935-s001.zip › cancers-3248027-supplementary.pdf]

Search Strategies

MEDLINE via PubMed was searched on September 2023, using the search strategy: ((#1 AND #2 AND #3) NOT #4) NOT #5 where the numbers represent the following search terms:

#1. ("Soft Tissue Neoplasms"[Mesh] OR (("neoplasm"[TIAB] OR "neoplasms"[TIAB] OR "tumor"[TIAB] OR "tumour"[TIAB] OR "tumors"[TIAB] OR "tumours"[TIAB]) AND “soft tissue”[TIAB]))

#2. ("referral and consultation"[MeSH Terms] OR "referral"[TIAB] OR "referrals"[TIAB] OR "referrer"[TIAB] OR "referrers"[TIAB])

#3. ("Diagnostic Imaging"[Mesh] OR "Magnetic resonance imaging"[TIAB] OR "MRI"[TIAB] OR "Ultrasonography"[TIAB])

#4. ("case reports"[Publication type] OR "case report"[All Fields] OR "case-series"[All Fields])

#5. ('adeno*'[TIAB] OR 'hemangio*'[TIAB] OR 'retroperitoneal'[TIAB] OR 'paget'[TIAB] OR 'angiosarcoma'[TIAB] OR 'rhabdomyosarcoma'[TIAB] OR 'myxo*'[TIAB] OR 'carcino*'[TIAB] OR 'leukemia'[TIAB] OR 'lymphoma'[TIAB] OR 'synovial'[TIAB] OR 'desmoid'[TIAB] OR 'rheum*'[TIAB] OR 'melanoma'[TIAB] OR 'hand'[TIAB] OR 'chordoma'[TIAB] OR 'ankle'[TIAB] OR 'breast'[TIAB] OR 'bladder'[TIAB] OR 'head'[TIAB] OR 'feline'[TIAB] OR 'amputation'[TIAB] OR 'histio*'[TIAB] OR 'ewing'[TIAB] OR 'patho*'[TIAB] OR 'gene'[TIAB] OR 'colorec*'[TIAB] OR 'meningioma'[TIAB] OR 'renal'[TIAB] OR 'dogs'[TIAB] OR 'cat*'[TIAB] OR 'bone tumor*'[TIAB] OR 'osteoid'[TIAB] OR 'excision'[TIAB] OR 'haemangioma'[TIAB] OR 'leiomyo*'[TIAB] OR 'kaposi'[TIAB] OR 'gist'[TIAB] OR 'pneum*'[TIAB] OR 'gynec*'[TIAB] OR 'gynaec*'[TIAB] OR 'report'[TIAB] OR 'gastr*'[TIAB] OR 'prosta*'[TIAB] OR 'pseudo*'[TIAB] OR 'uter*'[TIAB] OR 'menstr*'[TIAB] OR 'cardi*'[TIAB] OR 'race'[TIAB] OR 'robot*'[TIAB] OR 'lymph*'[TIAB] OR 'pancr*'[TIAB] OR 'hyster*'[TIAB] OR 'fibro*'[TIAB] OR 'covid'[TIAB] OR 'osteoma'[TIAB] OR 'chondro*'[TIAB] OR 'hair*'[TIAB] OR 'ocula*'[TIAB] OR 'nephro*'[TIAB] OR 'hepat*'[TIAB] OR 'HIV'[TIAB] OR 'retin*'[TIAB] OR 'nose'[TIAB] OR 'ear'[TIAB] OR 'nose'[TIAB] OR 'syphilis'[TIAB] OR ‘lipom*'[TIAB] OR 'syndro*'[TIAB] OR ‘derma*'[TIAB] OR 'cochlea*'[TIAB] OR 'neur*'[TIAB])

The search was restricted to articles written in English.

EMBASE was searched on September 2023, using the search strategy: ((#1 AND #2 AND #3) NOT #4) where the numbers represent the following search terms:

#1. 'soft tissue tumor'/exp OR (("neoplasm":ab,ti,kw OR "neoplasms":ab,ti,kw OR "tumor":ab,ti,kw OR "tumour":ab,ti,kw OR "tumors":ab,ti,kw OR "tumours":ab,ti,kw) AND “soft tissue”:ab,ti,kw)

#2. 'patient referral'/exp OR ('referral':ab,ti,kw OR 'referrals':ab,ti,kw OR

'referrer*':ab,ti,kw OR 'referrers':ab,ti,kw)

#3. ('nuclear magnetic resonance imaging'/exp OR 'Magnetic resonance imaging':ab,ti,kw OR 'MRI':ab,ti,kw) OR ('echography'/exp OR 'echo*':ab,ti,kw OR 'ultrasound':ab,ti,kw)

#4. 'case*':ab,ti,kw OR 'edit*':ab,ti,kw OR 'report':ab,ti,kw OR 'adeno*':ab,ti,kw OR 'hemangio*':ab,ti,kw OR 'retroperitoneal':ab,ti,kw OR 'paget':ab,ti,kw OR 'angiosarcoma':ab,ti,kw OR 'rhabdomyosarcoma':ab,ti,kw OR 'myxo*':ab,ti,kw OR 'carcino*':ab,ti,kw OR 'leukemia':ab,ti,kw OR 'lymphoma':ab,ti,kw OR 'synovial':ab,ti,kw OR 'desmoid':ab,ti,kw OR 'rheum*':ab,ti,kw OR 'melanoma':ab,ti,kw OR 'hand':ab,ti,kw OR 'chordoma':ab,ti,kw OR 'ankle':ab,ti,kw OR 'breast':ab,ti,kw OR 'bladder':ab,ti,kw OR 'head':ab,ti,kw OR 'feline':ab,ti,kw OR 'amputation':ab,ti,kw OR 'histio*':ab,ti,kw OR 'ewing':ab,ti,kw OR 'patho*':ab,ti,kw OR 'gene':ab,ti,kw OR 'colorec*':ab,ti,kw OR 'meningioma':ab,ti,kw OR 'renal':ab,ti,kw OR 'dogs':ab,ti,kw OR 'cat*':ab,ti,kw OR 'bone tumor*':ab,ti,kw OR 'osteoid':ab,ti,kw OR 'excision':ab,ti,kw OR 'haemangioma':ab,ti,kw OR 'leiomyo*':ab,ti,kw OR 'kaposi':ab,ti,kw OR 'gist':ab,ti,kw OR 'pneum*':ab,ti,kw OR 'gynec*':ab,ti,kw OR 'gynaec*':ab,ti,kw OR 'report':ab,ti,kw OR 'gastr*':ab,ti,kw OR 'prosta*':ab,ti,kw OR 'pseudo*':ab,ti,kw OR 'uter*':ab,ti,kw OR 'menstr*':ab,ti,kw OR 'cardi*':ab,ti,kw OR 'race':ab,ti,kw OR 'robot*':ab,ti,kw OR 'lymph*':ab,ti,kw OR 'pancr*':ab,ti,kw OR 'hyster*':ab,ti,kw OR 'fibro*':ab,ti,kw OR 'covid':ab,ti,kw OR 'osteoma':ab,ti,kw OR 'chondro*':ab,ti,kw OR 'hair*':ab,ti,kw OR 'ocula*':ab,ti,kw OR 'nephro*':ab,ti,kw OR 'hepat*':ab,ti,kw OR 'HIV':ab,ti,kw OR 'retin*':ab,ti,kw OR 'nose':ab,ti,kw OR 'ear':ab,ti,kw OR 'nose':ab,ti,kw OR 'syphilis':ab,ti,kw OR ‘lipom*':ab,ti,kw OR 'syndro*':ab,ti,kw OR ‘derma*':ab,ti,kw OR 'cochlea*':ab,ti,kw OR 'neur*':ab,ti,kw

The search was restricted to articles written in English.

Cochrane library was searched on September 2023, using the search strategy: #1 AND #2 AND #3 NOT #4 where the numbers represent the following search terms:

#1. [mh "Soft Tissue Neoplasms"] OR (('neoplasm*':ab,ti,kw OR 'tumor*':ab,ti,kw OR 'tumour*':ab,ti,kw) AND ('soft tissue':ab,ti,kw))

#2. [mh “Referral and Consultation”] OR ('referral*':ab,ti,kw OR 'referrer*':ab,ti,kw)

#3 [mh “Diagnostic Imaging”] OR 'Magnetic resonance imaging':ab,ti,kw OR 'MRI':ab,ti,kw OR 'echo*':ab,ti,kw OR 'ultrasound':ab,ti,kw

#4 'case*':ab,ti,kw OR 'edit*':ab,ti,kw OR 'report':ab,ti,kw OR 'adeno*':ab,ti,kw OR 'hemangio*':ab,ti,kw OR 'retroperitoneal':ab,ti,kw OR 'paget':ab,ti,kw OR 'angiosarcoma':ab,ti,kw OR 'rhabdomyosarcoma':ab,ti,kw OR 'myxo*':ab,ti,kw OR 'carcino*':ab,ti,kw OR 'leukemia':ab,ti,kw OR 'lymphoma':ab,ti,kw OR 'synovial':ab,ti,kw OR 'desmoid':ab,ti,kw OR 'rheum*':ab,ti,kw OR 'melanoma':ab,ti,kw OR 'hand':ab,ti,kw OR 'chordoma':ab,ti,kw OR 'ankle':ab,ti,kw OR 'breast':ab,ti,kw OR 'bladder':ab,ti,kw OR 'head':ab,ti,kw OR 'feline':ab,ti,kw OR 'amputation':ab,ti,kw OR 'histio*':ab,ti,kw OR 'ewing':ab,ti,kw OR 'patho*':ab,ti,kw OR 'gene':ab,ti,kw OR

'colorec*':ab,ti,kw OR 'meningioma':ab,ti,kw OR 'renal':ab,ti,kw OR 'dogs':ab,ti,kw OR 'cat*':ab,ti,kw OR 'bone tumor*':ab,ti,kw OR 'osteoid':ab,ti,kw OR 'excision':ab,ti,kw OR 'haemangioma':ab,ti,kw OR 'leiomyo*':ab,ti,kw OR 'kaposi':ab,ti,kw OR 'gist':ab,ti,kw OR 'pneum*':ab,ti,kw OR 'gynec*':ab,ti,kw OR 'gynaec*':ab,ti,kw OR 'report':ab,ti,kw OR 'gastr*':ab,ti,kw OR 'prosta*':ab,ti,kw OR 'pseudo*':ab,ti,kw OR 'uter*':ab,ti,kw OR 'menstr*':ab,ti,kw OR 'cardi*':ab,ti,kw OR 'race':ab,ti,kw OR 'robot*':ab,ti,kw OR 'lymph*':ab,ti,kw OR 'pancr*':ab,ti,kw OR 'hyster*':ab,ti,kw OR 'fibro*':ab,ti,kw OR 'covid':ab,ti,kw OR 'osteoma':ab,ti,kw OR 'chondro*':ab,ti,kw OR 'hair*':ab,ti,kw OR 'ocula*':ab,ti,kw OR 'nephro*':ab,ti,kw OR 'hepat*':ab,ti,kw OR 'HIV':ab,ti,kw OR 'retin*':ab,ti,kw OR 'nose':ab,ti,kw OR 'ear':ab,ti,kw OR 'nose':ab,ti,kw OR 'syphilis':ab,ti,kw OR ‘lipom*':ab,ti,kw OR 'syndro*':ab,ti,kw OR ‘derma*':ab,ti,kw OR 'cochlea*':ab,ti,kw OR 'neur*':ab,ti,kw
